# Evolution of a CD3^+^/CD^+^ *α/ β* T-Cell Receptor^+^ Mature T-Cell Clone from CD3^-^CD7^+^ Sorted Human Bone Marrow Cells

**DOI:** 10.1155/1993/59852

**Published:** 1993

**Authors:** Heike Pohla, Medi Adibzadeh, Hans-Jörg Bühring, Petra Siegels-Hübenthal, Thomas Deikeler, Martin Owsianowsky, Andrea Schenk, Arnika Rehbein, Elke Schlotz, Kurt Schaudt, Graham Pawelec

**Affiliations:** 1 Medical and Natural Sciences Research Center of the University of Tübingen Tübingen University Medical Clinic Tübingen D-7400 Germany; 2 Section for Transplantation Immunology and Immunohematology Tübingen University Medical Clinic Tübingen D-7400 Germany; 3 Division of Immunopathology Second Department of Internal Medicine Tübingen University Medical Clinic Tübingen D-7400 Germany; 4 Department of Dermatology Klinikum Steglitz Berlin Germany; 5 Abt. Innere Medizin II Medizinische Universitätsklinik und Poliklinik Tübingen D-7400 Germany

**Keywords:** T-cell development, interleukin-2, bone marrow, T-cell precursors, extrathymic differentiation

## Abstract

In order to study extrathymic differentiation *in vitro*, CD7^+^CD3^-^ lymphocytes were sorted from normal human bone marrow and cultured under conditions of limiting dilution together with irradiated pooled allogeneic peripheral blood mononuclear cells (PBMC) and phytohemagglutinin (PHA) in the presence of 1000 U/ml of interleukin-2 (IL-2). One clone was obtained that failed to react with monoclonal antibody (mAb) TCR*δ*1 (TCR*γ/δ*-specific) or WT31 (TCR2, *α/β*-specific). From day 35 through day 74 in culture, the surface phenotype of this clone evolved into CD3^+^, CD4^+^, CD8^-^, TCR2^+^, TCR1^-^, and was further characterized as CD2^+^, CD45RO^+^, CD16^-^, and CD56^-^. The presence of mRNA for TCR *α* and *γ* but not ,and *γ* chains was confirmed by Northern blotting. Accessory cell-dependent autocrine proliferative responses to PHA (most likely driven by IL-2) were initially absent, but became measurable at the same time as the TCR was acquired. However, in the absence of PHA, the clone failed to respond to a panel of homozygous B-cell lines representing the majority of MHC class II alleles. Autoreactivity was also not demonstrable. Cytotoxicity was limited to MHC unrestricted “natural killer (NK)-like” lysis of K562 target cells, with no autocytotoxicity detected. Tle NK-like lysis diminished over time in parallel with the acquisition of surface TCR. The cloned cells were not suppressive for mature lymphocyte proliferation. After stimulation, the cells secreted tumor necrosis factor *α* and granulocyte/macrophage colony-stimulating factor (GM-CSF) detected by immunoassays, and T-cell growth factors, most likely IL-2, as detected by bioassays. Polymerase chain-reaction methods demonstrated the presence of mRNA for IL-2, IL-3, IL-4, IL-9, interferon-*δ*, and GM-CSF in these cells after stimulation with PHA and B-LCL.

These results suggest that cells with the phenotype and some functional characteristics of mature T lymphocytes can evolve extrathymically *in vitro* from T-cell precursors sorted from normal human bone marrow.


*Developmental Immunology*, 1993, Vol. 3, pp. 197–210
Reprints available directly from the publisher
Photocopying permitted by license only

© 1993 Harwood Academic Publishers GmbH
Printed in the United States of America

### Evolution of a $CD3^+CD4^+$ $\alpha/\beta$ T-Cell Receptor$^+$ Mature T-Cell Clone from $CD3^-CD7^+$ Sorted Human Bone Marrow Cells

HEIKE POHLA,† MEDI ADIBZADEH,† HANS-JÖRG BÜHRING,‡ PETRA SIEGELS-HÜBENTHAL,† THOMAS DEIKELER,† MARTIN OWSIANOWSKY,§|| ANDREA SCHENK,† ARNIKA REHBEIN,‡ ELKE SCHLOTZ,‡ KURT SCHAUDT,‡ and GRAHAM PAWELEC*†‡

†*Medical and Natural Sciences Research Center of the University of Tübingen,*
‡*Section for Transplantation Immunology and Immunohematology,*
§*Division of Immunopathology, Second Department of Internal Medicine, Tübingen University Medical Clinic, D-7400 Tübingen, Federal Republic of Germany*

In order to study extrathymic differentiation *in vitro*, $CD7^+CD3^-$ lymphocytes were sorted from normal human bone marrow and cultured under conditions of limiting dilution together with irradiated pooled allogeneic peripheral blood mononuclear cells (PBMC) and phytohemagglutinin (PHA) in the presence of 1000 U/ml of interleukin-2 (IL-2). One clone was obtained that failed to react with monoclonal antibody (mAb) TCR$\delta$1 (TCR1, $\gamma/\delta$-specific) or WT31 (TCR2, $\alpha/\beta$-specific). From day 35 through day 74 in culture, the surface phenotype of this clone evolved into $CD3^+$, $CD4^+$, $CD8^-$, $TCR2^+$, $TCR1^-$, and was further characterized as $CD2^+$, $CD45RO^+$, $CD16^-$, and $CD56^-$. The presence of mRNA for TCR $\alpha$ and $\beta$ but not $\gamma$ and $\delta$ chains was confirmed by Northern blotting. Accessory cell-dependent autocrine proliferative responses to PHA (most likely driven by IL-2) were initially absent, but became measurable at the same time as the TCR was acquired. However, in the absence of PHA, the clone failed to respond to a panel of homozygous B-cell lines representing the majority of MHC class II alleles. Autoreactivity was also not demonstrable. Cytotoxicity was limited to MHC unrestricted "natural killer (NK)-like" lysis of K562 target cells, with no autocytotoxicity detected. The NK-like lysis diminished over time in parallel with the acquisition of surface TCR. The cloned cells were not suppressive for mature lymphocyte proliferation. After stimulation, the cells secreted tumor necrosis factor $\alpha$ and granulocyte/macrophage colony-stimulating factor (GM-CSF) detected by immunoassays, and T-cell growth factors, most likely IL-2, as detected by bioassays. Polymerase chain-reaction methods demonstrated the presence of mRNA for IL-2, IL-3, IL-4, IL-9, interferon-$\gamma$, and GM-CSF in these cells after stimulation with PHA and B-LCL.

These results suggest that cells with the phenotype and some functional characteristics of mature T lymphocytes can evolve extrathymically *in vitro* from T-cell precursors sorted from normal human bone marrow.

KEYWORDS: T-cell development; interleukin-2; bone marrow; T-cell precursors; extrathymic differentiation.

### INTRODUCTION

T-cell precursors arise in the bone marrow, from where they must migrate to the thymus via peripheral blood. That the thymus is important for the generation of the mature T lymphocytes, for which both positive and negative selection of developing T cells is required, is demonstrated by their virtual absence in young congenitally athymic rats, mice, and di George Syndrome patients. However, it is not clear whether the thymus is absolutely necessary for the T-cell differentiation process, because older athymic rodents do possess limited numbers of both TCR1 ($\gamma/\delta$-

||Present address: Department of Dermatology, Klinikum Steglitz, Berlin.
*Corresponding author. Present address: Abt. Innere Medizin II, Medizinische Universitätsklinik und Poliklinik, D-7400 Tübingen, Federal Republic of Germany.

receptor$^+$) and TCR2 ($\alpha/\beta$-receptor$^+$) T lymphocytes (Benveniste et al., 1990; Sarawar et al., 1991). Moreover, analysis of such cells after long-term alloantigen-stimulated culture indicated the presence of functional cytokine-secreting autoreactive and alloreactive T cells (Abromson-Leeman et al., 1990), although *in vivo* function may nonetheless be severely compromised (Sarawar et al., 1991). Taken together, these results suggest that extrathymic T-cell generation resulting in functionally competent cells may occur, but the possibility remained that even athymic rodents might retain thymic rudiments that could influence T-cell maturation.

Several lines of evidence suggest that extrathymic maturation may also occur in man. Early data suggested that peripheral blood mononuclear cells (PBMC) stringently depleted of T cells formed colonies that rapidly acquired surface CD3 in semisolid agar cultures (Triebel et al., 1981) and in liquid cultures under limiting dilution conditions (Moreau and Miller, 1983). Peripheral lymphocytes expressing an early T-cell marker, CD7, but lacking the T-cell receptor (TCR) for antigen, were found slowly to acquire CD3 and TCR when cultured with interleukin (IL-2), phytohemagglutinin (PHA), and allogeneic cells (Preffer et al., 1989). However, it was pointed out that it could not be excluded that these peripheral cells had been exposed to the thymus, but had "escaped" without processing. To exclude this, Hori and co-workers (1991) cultured CD3$^-$ human fetal liver cells with PHA and allogeneic cells and reported the generation of T cells with both mature (surface CD3$^+$) as well as a variety of immature (cytoplasmic CD3$^+$) phenotypes. In another series of studies by a different group, cells of the same phenotype derived from bone marrow (BM) cultured with PHA-conditioned medium began to acquire CD3 as early as day 3 (Bertho et al., 1990). In a different approach, CD7$^+$CD3$^-$ human BM cells cultured in agar were shown to form colonies expressing CD3 after only a week (Lecron et al., 1989), provided that cultures were supplemented with factors additional to IL-2 (Mossalayi et al., 1985). Taken together, these and other results suggest that pre-T cells in BM and possibly also PBMC can develop extrathymically into cells carrying the phenotypes of mature thymic-processed T lymphocytes. It might be predicted that such cells would differ from postthymic clones in their functions and specificities, particularly autoreactivity. So far, this appears not to have been investigated. We therefore undertook a study on the functional characteristics of T-cell clones (TCC) generated from CD7$^+$CD3$^-$ cells in normal BM.

### RESULTS

### Surface-Marker Phenotyping and Nature of the TCR

CD3$^-$CD7$^+$ BM cells from two healthy donors were cloned by limiting dilution in the presence of allogeneic PBMC, PHA, and IL-2. Cloning efficiencies were 17% and 18%, yielding 19 and 17 T-cell clones, respectively. The surface phenotypes of these TCC were established at the earliest time point possible (days 23 to 35). With one exception, all clones stained strongly with mAb OKT3 and WT31 (data not shown). Most of these were not studied further here. One clone, designated 314-14, failed to stain with mAb WT31 and stained only weakly with OKT3 at the earliest time point tested (day 35). At the next time point tested, culture day 56, the phenotype had changed radically. About half the cells had acquired bright staining with WT31, and there was a separate peak of less strongly staining cells as well as a small population that remained completely negative (Fig. 1). The pattern of CD3 expression paralleled that of WT31 (Fig. 1) and there was no staining with mAb TCR$\delta$1 (not shown). By day 65, almost all cells stained brightly with both OKT3 and WT31, but there was still a small population of more weakly staining cells. This had completely disappeared by day 74 (Fig. 1). Cells were also tested with mAb to polymorphic determinants carried by TCR V$\beta$ chains at days 56 and 73, but failed to stain with V$\beta$ 5, 6 or 8-specific mAb (data not shown). The cells stained homogeneously brightly with CD2 and CD7 mAb at all four time points tested (data not shown). Because 314-14 was unique in appearance and behavior, the question of its origin arose (abnormal mutated cell derived from irradiated filler cells?). Therefore, PBMC from the donor, clone 314-14 and another T-cell clone from the same donor were subjected to HLA class I serotyping using complement previously absorbed with other T-cell clones from the same individual. They all

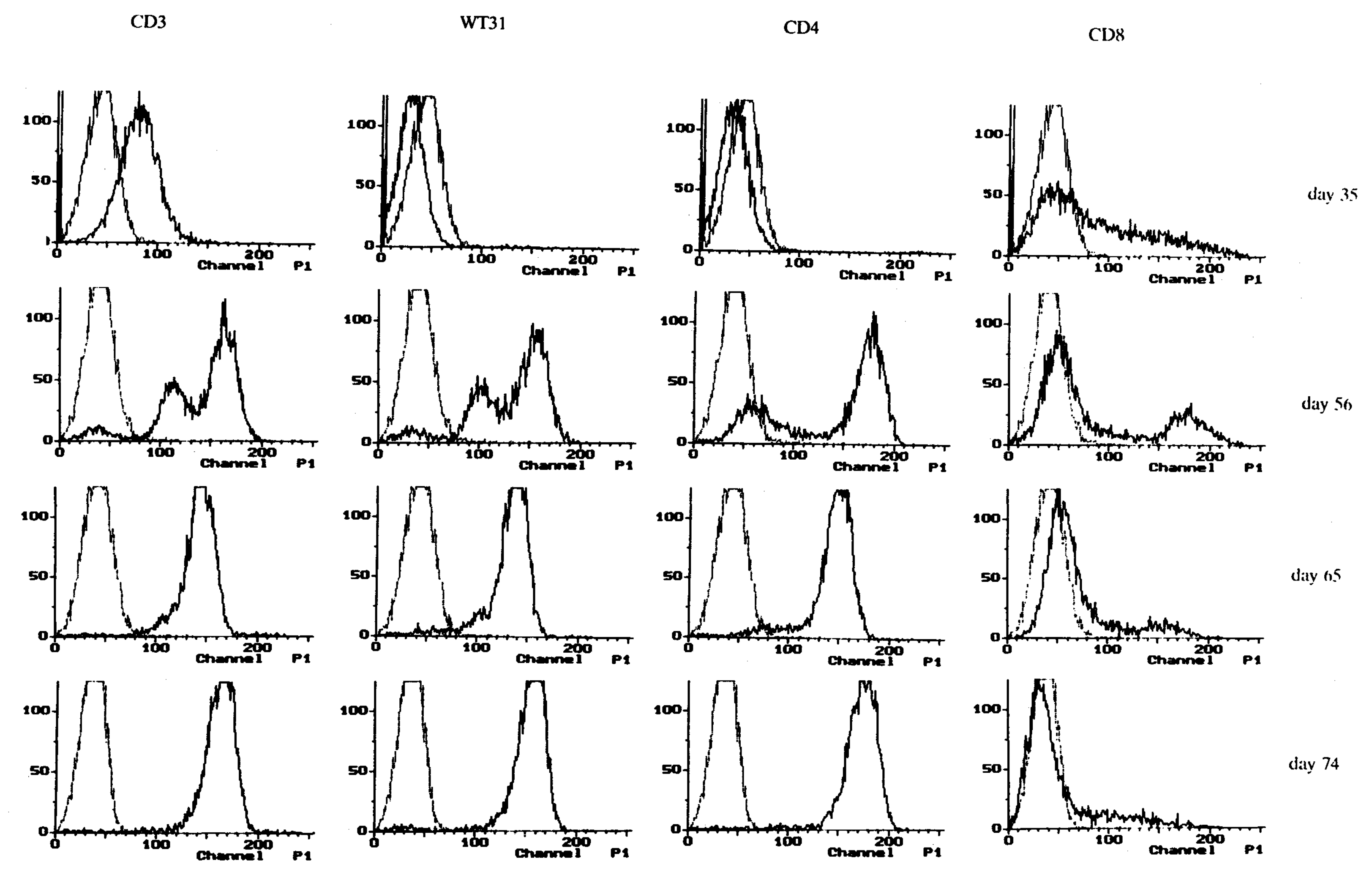

FIGURE 1. Evolution in culture of the cell-surface phenotype of clone 314-14. The $y$-axis depicts the number of cells per channel analyzed; the $x$-axis depicts the arbitrary fluorescence intensity.

expressed an identical HLA-A1, 25; -B8, 61; -Cw2, Cw7 phenotype (data not shown) and therefore can be taken as originating from the same donor.

The evolution of the CD4/8 phenotype of clone 314-14 was also followed. Initially, the clone expressed very little if any CD4, but did express CD8 in a continuously variable manner (Fig. 1). By day 56, a separate bright peak and smaller negative peak for CD4 became visible, with CD8 staining resulting in a reciprocal picture. The level of CD8 staining became progressively less, until by day 74, the clone was essentially $CD4^+CD8^-$ (Fig. 1).

Both at day 56 and day 73, clone 314-14 cells strongly expressed the CD45RO isoform of the leukocyte common antigen (median channel intensity 169 and 122, respectively, compared with background staining of 33 and 34, respectively), but also expressed CD45RA (albeit at the lower levels of 90 and 70, respectively).

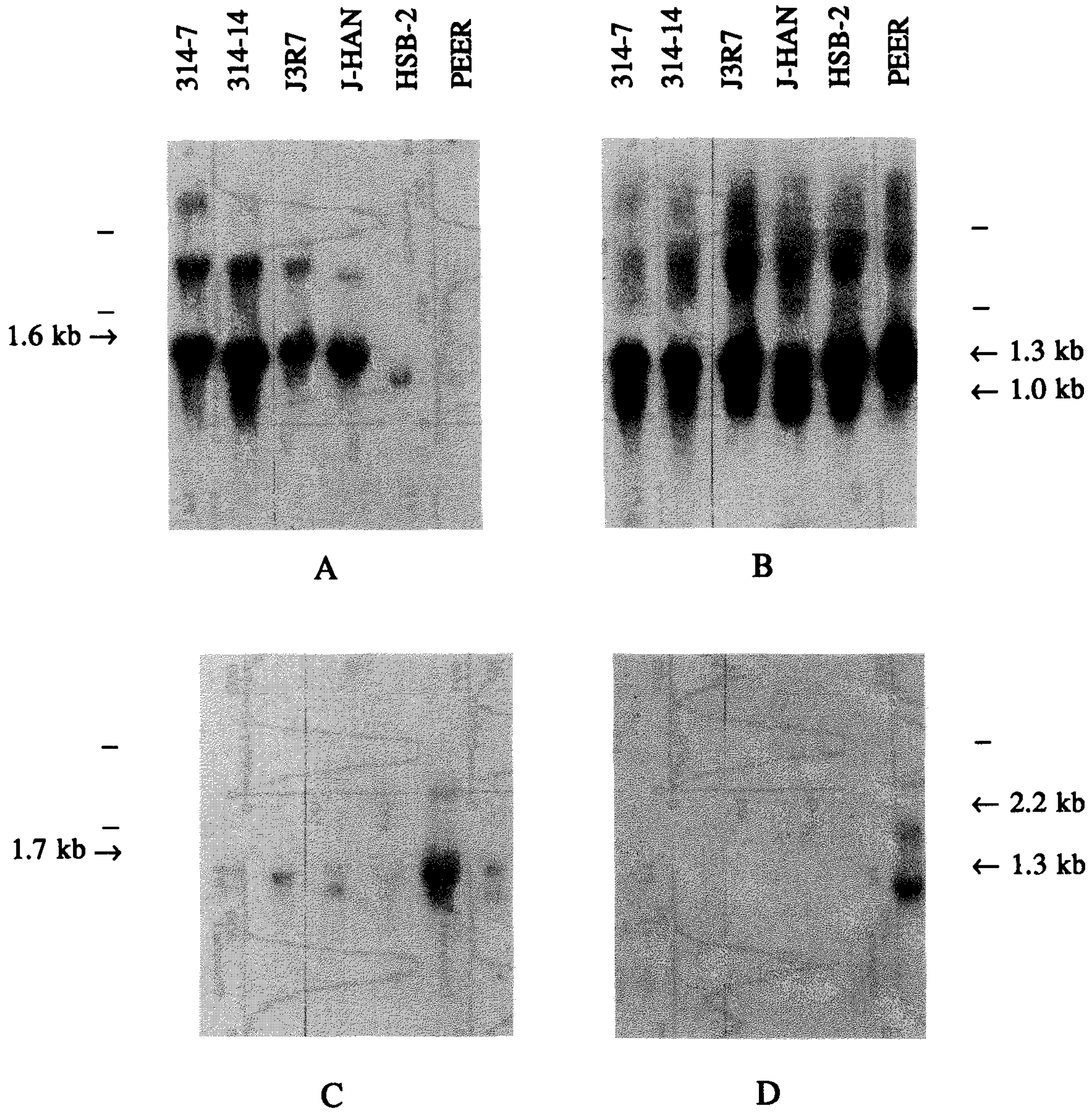

FIGURE 2. Northern blot analysis TCC 314-7 and 314-14 TCR mRNA. Hybridization was done with the TCR-$\alpha$-chain-specific probe pGA5 (A), the TCR$\beta$-chain probe pC$\beta$REX (B), and with probes specific for TCR$\gamma$ (C) and TCR$\delta$ (D). Controls were JURKAT T-ALL lines J3R7 and JHAN, and the immature T-cell lines HSB2 and PEER. The 18 and 28S rRNAs are indicated by the two dashes.

They stained weakly for CD5 and CD25 (ca. 50 and 60 arbitrary units, respectively), and did not express CD16 or CD56 (background staining).

The expression of mRNA for TCR $\alpha$ and $\beta$ chains was confirmed using Northern blotting in 65-day-old cells. Figure 2 compares clone 314-14 with another clone from the same donor, 314-7, which had already demonstrated a mature phenotype at the earliest time point tested. The same two clones were assessed in Southern blotting at the same age for TCR gene rearrangements. The result shown in Fig. 3 suggests monoallelic rearrangements involving C$\beta$1 and C$\beta$2 in clone 314-14, confirming monoclonality. Hybridization with the TCR$\gamma$ probe also indicated J$\gamma$1 and 2 rearrangements (data not shown).

### Proliferative Capacity of Clone 314-14: Response to Cytokines

Cells from clone 314-14 at culture days 56 and 74 were incubated together with IL-2, IL-3, IL-4, IL-7, or GM-CSF for 42, 66, or 90 hr before assessing proliferation by incorporation of $^3$H-TdR. The results for peak incorporation (66 hr) are shown in Fig. 4, suggesting that a major growth factor for these cells is IL-2.

### Proliferative Capacity of Clone 314-14: Response to Cells

The autocrine proliferative capacity of clone 314-14 cells was investigated by stimulating them with B-LCL and 1% PHA in the absence of exogenous cytokines. Thirty-five-day-old cells were unable to respond. Fifty-six-day-old cells responded very weakly, but 74-day-old cells proliferated strongly (Fig. 5). Thus, acquisition of autocrine proliferative capacity, triggered by the nonspecific mitogen PHA, parallels the acquisition of the TCR. However, stimulation by B-LCL in the absence of PHA did not result in autocrine proliferation (Fig. 5). An extended panel of stimulating B-LCL covering most class II alleles also failed to reveal alloreactivity of 314-14 cells (data not shown).

It was of interest to investigate whether, in the absence of the thymus, clone 314-14 cells had retained autoreactivity during differentiation. Stimulation of day 35, 56 and 74 cells with auto-

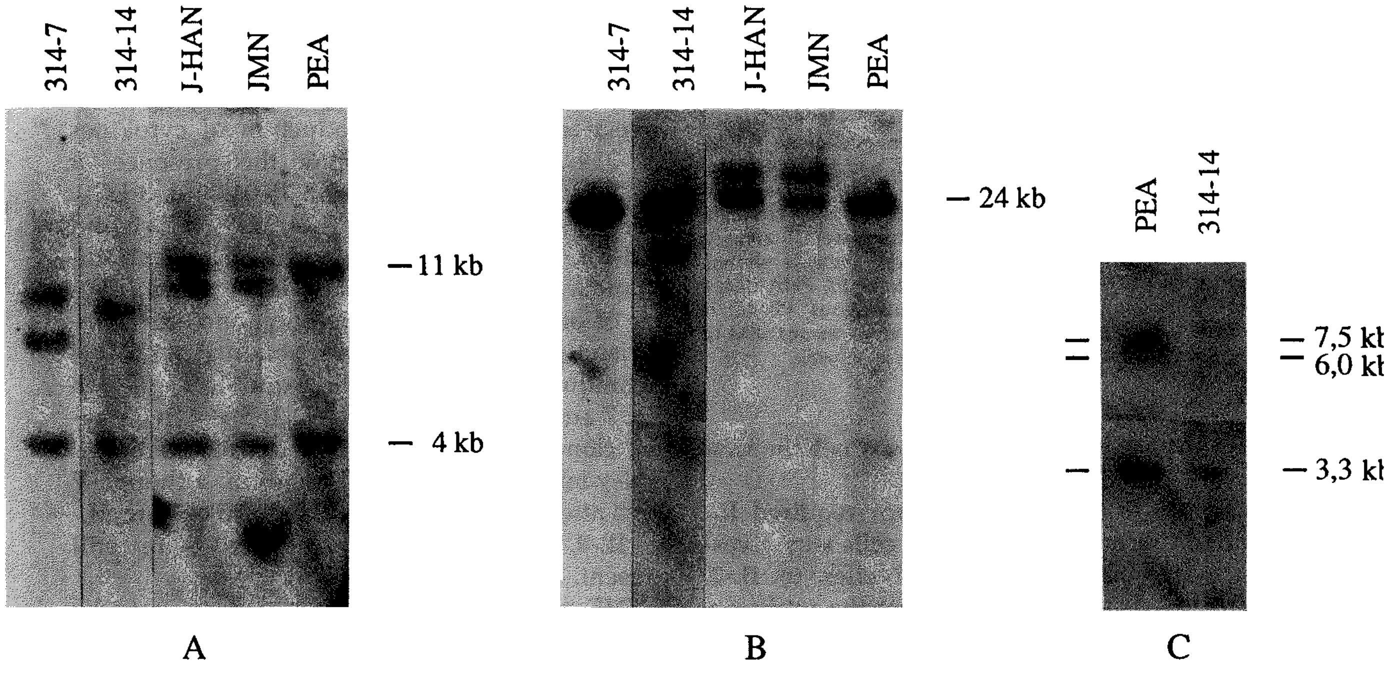

FIGURE 3. Southern blot analysis of (A) EcoRI-, (B) BamHI-, or (C) HindIII-digested genomic DNA isolated from the TCC 314-7 and 314-14, hybridized with the cDNA probe pC$\beta$REX. The B-LCL (PEA) represents the germline configuration of 11 kb (C$\beta$1 region) and 4 kb (C$\beta$2) for the EcoRI digest; 24 kb (C$\beta$1 and C$\beta$2) for the BamHI digest; and 7.5 kb (C$\beta$2), 6.0 kb (C$\beta$2 3' region), and 3.3 kb (C$\beta$1) for the HindIII digest. The JURKAT lines are included as positive controls for rearrangements on both alleles.

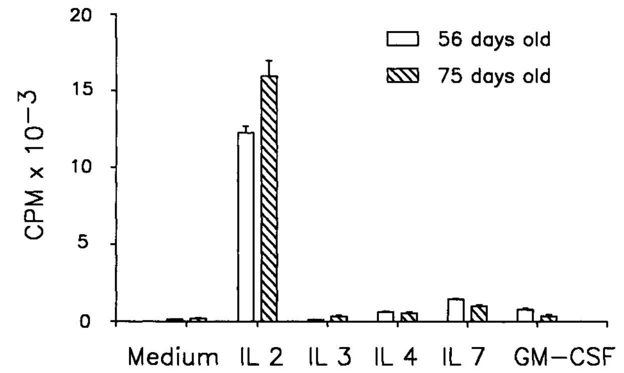

FIGURE 4. Proliferative responses of clone 314-14 to cytokines. The cytokines were titrated over a wide concentration range and a three time-point kinetic was performed. Maximal response is shown as cpm±SEM of triplicate cultures for IL-2 at 100 U/ml, IL-3 at 100 U/ml, IL-4 at 1000 U/ml, IL-7 at 50 U/ml, and GM-CSF at 50 U/ml, all after 66 hr. One of two experiments is shown.

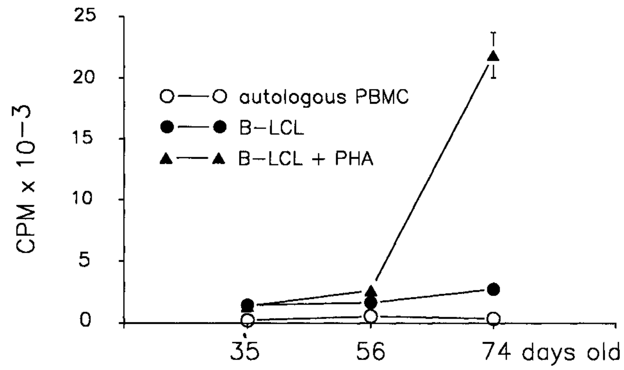

FIGURE 5. Autocrine proliferative responses of clone 314-14. A three time-point kinetic was performed, and the maximum responses are shown (obtained at 66 hr). A large panel of B-LCL (filled circles, n=46) representing essentially all common MHC alleles was employed as stimulators, and a representative result obtained with one of them is shown. Response to the same LCL in the presence of 1% PHA (filled triangles). Stimulation by irradiated autologous PBMC is represented by open circles. Data are given as mean±SEM of triplicates. One of four similar experiments is shown.

logous PBMC showed that this was not the case (Fig. 5).

The nature of the growth factor required for autocrine proliferation by 314-14 cells was investigated using neutralizing antibodies to IL-2. Data displayed in Fig. 6 show that the proliferative response of the cells to PHA in the presence of LCL accessory cells is inhibited by anti-IL-2 antibodies in a dose-dependent fashion, whereas isotype-matched anti-IL-1$\beta$ (Fig. 6) or anti-IL-1$\alpha$ (data not shown) had no effect on proliferation.

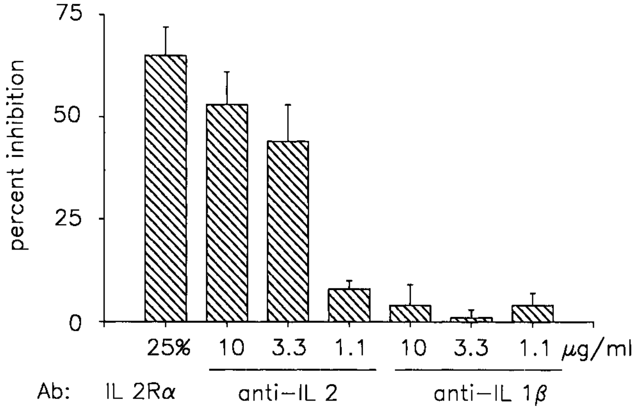

FIGURE 6. Inhibition of clone 314-14 autocrine proliferation with neutralizing IL-2 antibodies or CD25 mAb. Cells were stimulated with PHA in the presence of LCL and titrated amounts of IL-2 or IL-1$\beta$-specific purified IgG antibodies, or with 25% culture supernatant of the IL-2-$\alpha$-chain-specific mAb TÜ69. Results are expressed as percent inhibition±SEM of proliferation measured in the absence of antibody. One of two experiments is shown.

Moreover, an mAb against the $\alpha$ chain of the IL-2R also strongly blocked autocrine proliferation of 314-14 cells. Taken together, these data suggest that IL-2 is an important autocrine TCGF for clone 314-14 cells.

### Cytokine Production

A sensitive PCR technique was employed to investigate further the production of cytokines by 314-14 cells by assessing the presence of mRNA after stimulation with PHA and B-LCL. The presence of IL-2, IL-3, IL-4, IL-9, IFN-$\gamma$, and GM-CSF transcripts before and 5 hr after stimulation was assessed on clone 314-14 and a number of other TCC from the same donor for comparison. The kinds of results obtained with this technique are shown for the example of IL-9 in Fig. 7 and summarized in Table 1. Sixty-five-day-old clone 314-14 cells contained IL-2, IL-3, and IFN-$\gamma$ mRNA only after stimulation, although 7 days after the previous stimulation in culture, IL-4, IL-9, and GM-CSF transcripts were still present.

TNF-$\alpha$ mRNA production could not be measured by the technique described before, because the stimulating B-cell lines were all positive in PCR for this cytokine. Supernatants from cloned cells 48 hr after stimulation were therefore assessed for TNF-$\alpha$ content in an immunoassay.

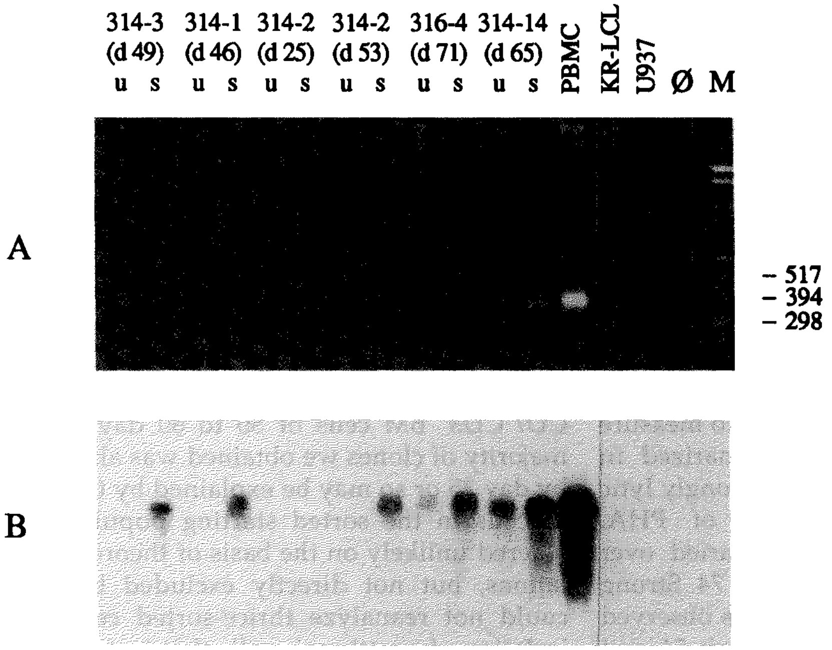

FIGURE 7. Southern blot analysis of the IL-9-specific amplified product of several TCC 7 days after stimulation in culture (u) or restimulated for 5 hr with 1% PHA and irradiated LCL. RNA from stimulated PBMC was used as a positive control. RNA from the human histiocytic lymphoma line U937, and additionally the performance of the amplification process without RNA served as negative controls. (A) Gel analysis of the amplified product (376 bp); M=molecular weight marker VI (Boehringer). (B) Hybridization with the IL-9-specific cDNA probe (kind gift of J. van Snick).

TABLE 1

Cytokine Production by Clone 314-14

| Clone | Stimulated | mRNA[a] | | | | | | Protein[b] | | Bioassay[c] |
|---|---|---|---|---|---|---|---|---|---|---|
| | | IL-2 | IL-3 | IL-4 | IL-9 | IFN-$\gamma$ | GM-CSF | GM-CSF | TNF-$\alpha$ | IL-2 |
| 314-14 | No | – | – | + | + | – | + | nt[d] | nt | nt |
| | Yes | + | + | + | + | + | + | 180 | 2025 | 68 |
| 314-3 | No | – | – | – | + | – | + | nt | nt | nt |
| | Yes | + | + | + | + | + | + | 71 | 750 | 45 |
| 314-12 | No | – | – | – | – | – | – | nt | nt | nt |
| | Yes | + | + | + | + | + | + | nt | nt | nt |
| 314-13 | No | – | – | – | – | – | – | nt | nt | nt |
| | Yes | + | + | + | + | + | + | 64 | 642 | 75 |
| 314-18 | No | – | – | – | – | – | – | nt | nt | nt |
| | Yes | + | + | + | + | + | + | nt | nt | nt |

[a]mRNA measured by PCR.

[b]Protein measured by ELISA.

[c]TCGF bioactivity measured on an IL-2-dependent T-cell clone.

[d]nt=not tested.

Cells from clone 314-14 were found to produce large amounts of TNF-$\alpha$ after stimulation. Additionally, the presence of GM-CSF protein in supernatants of stimulated 314-14 cells was demonstrated (Table 1), showing that the message at least for this cytokine was translated. T-cell growth-factor activity detected in a bioassay on a T-cell clone with weak or absent IL-4 reactivity, and therefore presumably predominantly IL-2, is also shown in Table 1. This result is consistent with the antibody neutralization experiment shown in Fig. 6.

### Cytotoxicity

Lytic activity of clone 314-14 cells was assessed on natural killer-susceptible K562 target cells in the presence of PHA (to measure cytotoxic potential) or absence of PHA (to measure MHC unrestricted "natural killerlike" cytotoxicity). In

TABLE 2
Cytotoxicity of Clone 314-14 at Different Ages

| Age (days) | Target cells | | |
|---|---|---|---|
| | K562 | K562+PHA | Autol T cells |
| 35 | 646[a] | nt[b] | nt |
| 56 | 41 | 4150 | <0.1 |
| 65 | <0.1 | 195 | <0.1 |
| 73 | <0.1 | 103 | <0.1 |
| 74 | <0.1 | 337 | <0.1 |

[a]Results given as mean lytic units (25%) per $10^7$ cells on 2000 targets (three experiments).

[b]nt=not tested.

addition, PHA-stimulated T-cell blasts of the clone donor were also used as targets to measure autoreactivity. The results are summarized in Table 2. 314-14 cells at day 35 were strongly lytic on K562 targets in the presence of PHA. Although the level of cytotoxicity varied over time, it remained present up until day 74. Strong NK activity of the clone, however, was observed only on day 35, was very weak at day 56 and thereafter was no longer seen. In contrast, autocytotoxic activity was not observed at any age (Table 2).

### DISCUSSION

CD7, a member of the Ig superfamily, is an early marker of the T-cell lineage, which is expressed on fetal lymphocytes prior to colonization of the thymus and prior to TCR$\beta$-chain gene rearrangement or expression of CD1, 2, or 3 (Lobach et al., 1985; Haynes et al., 1988). It is retained on mature cells, but its function is unknown. It may be involved in both positive and negative (Carrera et al., 1988; Emara et al., 1989; Costantinides et al., 1991; Jung et al., 1992; Shimizu et al., 1992) regulation of T-cell responses.

$CD7^+$ BM cells lacking surface expression of the T-cell antigen receptor-associated CD3 structures rapidly develop into $CD3^+$ colonies under appropriate culture conditions in semisolid media (Lecron et al., 1989). This process requires unidentified factors in addition to IL-2 (Mossalayi et al., 1985). Because at least the majority of these BM cells are unlikely to have experienced intrathymic passage, this system offers a model for extrathymic differentiation in man. However, the antigen-recognition repertoire and functions of this type of *in vitro* differentiated cell seem not to have been investigated.

We set out to test some of the basic functional attributes of T-cell clones derived from $CD7^+CD3^-$ BM cells using a liquid culture system. Previous work by Preffer and co-workers (1989) using cells of the same phenotype derived from peripheral blood had shown their slow (beginning at 40 days; complete by 85 days) acquisition of CD3 positivity in uncloned lines in liquid culture using high concentrations of recombinant IL-2. The kinetics of acquisition of TCR by cloned cells was not specified (Preffer et al., 1989). Our results on clone 314-14 suggest a similar TCR acquisition kinetic by at least some $CD7^+CD3^-$ BM cells of 50 to 60 days. That the majority of clones we obtained was already $CD3^+$ by day 25 or so may be explained by (1) impurity of cells in the sorted starting population, considered unlikely on the basis of theoretical calculations, but not directly excluded because we could not reanalyze thrice-sorted cells; (2) the isolation of postthymic cells that had returned to the BM, or were contaminating it, and that had for some reason modulated CD3/TCR at the time of isolation; (3) the existence of a second type of extrathymic differentiation pathway where the TCR is acquired much more rapidly. The last possibility is consistent with results obtained using BM T-cell colony formation in agar (Triebel et al., 1981; Mossalayi et al., 1985; Hallet et al., 1989; Lecron et al., 1989; Mossalayi et al., 1990) and liquid culture (Moreau and Miller, 1983), but inconsistent with the PBMC-liquid culture system of Preffer and co-workers (1989). Moreover, recent results from Palathumpat and co-workers (1992) showed that sorted mouse $CD4^-CD8^-TCR2^-$ BM cells, which manifested germline configurations for TCR-$\beta$-chain genes, very rapidly acquired TCR2 expression in culture after only 2 days, and that this was accompanied by rearrangements in the $\beta$-chain genes.

We present for the first time functional data on cells with an apparently mature $CD2^+CD3^+$ $CD4^+CD7^+CD8^-TCR2^+$ phenotype derived from putative BM prethymic precursor T cells. These cells were clearly capable of PHA-stimulated autocrine proliferation once they had acquired a mature TCR phenotype (strongly $CD3^+WT31^+$), but not beforehand, even though PHA can trigger cell proliferation via structures other than exclusively by TCR (O'Flynn et al., 1985; Pantaleo et al., 1988). Thus, such cells acquire proliferative competence in parallel with the acquisition of the

TCR. In contrast, it appeared that they lost NK-like activity also in parallel with the acquisition of the TCR, although their cytotoxic potential was maintained. At no time point was it possible to demonstrate autoreactivity, either in proliferation or cytotoxicity assays. Furthermore, no alloreactivity could be demonstrated in either test system. These results may suggest: (1) the single clone 314-14 happens to be specific for a particular antigen for which we did not test, or (2) the differentiation of T cells in an environment containing most or all MHC types (on the pooled stimulators), plus PHA, leads to the deletion of precursors potentially recognizing them and allows only rare cells like 314-14 to escape deletion. It is also important to note that we were unable to show suppressive activity of these cells in MLC, a property that distinguishes them from the "natural suppressor" cells from BM, even those that have acquired TCR expression (Palathumpat et al., 1992).

Autocrine proliferative responses by 314-14 cells probably depended upon IL-2 as a growth factor, because (1) IL-2 mRNA was present in stimulated cells; (2) T-cell growth-factor activity was secreted by the cells; (3) the cells responded strongly to IL-2 but weakly or not at all to IL-4 and IL-7; and (4) PHA-stimulated autocrine proliferation was substantially inhibited by anti-IL-2 and CD25 antibodies. However, it is not excluded that IL-9 or IL-12 could act as growth factors for these cells, particularly because IL-2 neutralization did not result in complete suppression of proliferation and because we demonstrated the presence of IL-9 mRNA transcripts in 314-14 cells.

Cells from clone 314-14 were unique amongst clones from the same donor (Table 1) or alloreactive cells from other donors (data not shown) in being the only ones to express the IL-4 message without restimulation (i.e., 7 days after stimulation). It has been reported that human pro-T cells can synthesize IL-4 mRNA in the absence of an exogenous stimulus (Barcena et al., 1991), although it was suggested that these are destined to become TCR1$^+$ cells, whereas 314-14 possesses TCR2. However, some CD7$^+$CD3$^-$ human BM cells are clearly able to differentiate into TCR2 T cells *in vitro* under the influence of IL-2 combined with another unidentified factor (Bertho et al., 1990), and 314-14 may therefore have developed along this pathway. The presence of IL-2 may favor the development of TCR2$^+$ cells, whereas IL-4 or IL-7, not added to our culture system, may favor the differentiation of TCR1$^+$ cells (Barcena et al., 1991; Watanabe et al., 1991).

These results suggest that the approach presented here may represent a viable model for studies of human T-cell differentiation *in vitro*. However, it must be emphasized that the present culture technique resulted in the isolation of only a single clone, the differentiation of which could be followed in this way *in vitro*, and that this therefore may not necessarily be representative of extrathymic differentiation pathways. Further work aimed at improving the yield of such clones is required to resolve this question. Nonetheless, the ability to use this technique to explore possibilities to manipulate extrathymic differentiation of BM-derived T-cell precursors may be useful in the context of marrow transplantation in adults, where patients commonly possess minimal thymic function due to conditioning and age. Both long-lasting depressed T-cell function and manifestations of autoimmunity in chronic graft versus host disease may be reflections of inefficient thymic function, and be susceptible to manipulations aimed at influencing extrathymic differentiation.

### MATERIALS AND METHODS

### Cell Separations

Prior to staining, 4×10$^7$ BMMC were preincubated with human gamma-globulin (Intraglobin, Biotest, Frankfurt) for 10 min. After washing, cells were incubated with OKT3 (ATCC, CD3, IgG2a) and TÜ 93 (Tübingen local, CD7, IgG2b) for 15 min. After repeated washing, the cells were incubated with goat anti-mouse IgG2a-PE and goat anti-mouse IgG2b-FITC (Southern Biotechnology). Prior to sorting CD7$^+$CD3$^-$ cells with a FACS IV, a lymphocyte window was set by dual scatter parameters. The green fluorescence of FITC-labeled cells was measured through a 530-nm band-pass filter (Becton-Dickinson) and the yellow fluorescence of the PE-labeled antibodies through a 570-nm band-pass filter (Oriel). The yellow and green components of the emitted light were separated by a beam splitter with a dichroic (560-nm) filter. A

differential log amplifier was used to correct for the overlap of the green fluorescence emission spectrum of FITC into the yellow fluorescence emission spectrum of PE. The amplifier was adjusted so that the signal from cells stained only with FITC was orthogonal to the yellow fluorescence axis of the two-parameter yellow and green fluorescence display. For cell sorting, the sort window was set on the $CD7^+CD3^-$ population. One-droplet sorting without "full deflection envelope (coincidence)" was performed at a 20-kHz droplet formation rate (diameter of nozzle orifice was 80 $\mu$m). Cells were sorted into sterile tubes containing 20% FCS in Hanks' buffer and resorted twice in the same way. No $CD3^+$ cells could be found in 5000 reanalyzed cells from the $CD7^+CD3^-$ population. The enrichment factor for $CD3^-CD7^+$ cells at each sort was estimated at ×30, yielding a calculated purity of >99.9% after three sorts. There were too few cells available for restaining and reanalysis. The number of $CD3^-CD7^+$ cells initially present in the BM samples was estimated at ca. 0.5% rather more than reported previously (Bertho et al., 1990).

### Cell Culture

The $CD7^+CD3^-$ cells sorted from BM of two normal donors were cloned by limiting dilution in the presence of pooled allogeneic stimulator cells with 1000 U/ml IL-2 and PHA. Cells were plated at 0.45/well directly into Terasaki microplates containing $10^4$ 30 Gy-irradiated pooled PBMC from more than twenty random normal donors. Medium was RPMI 1640 supplemented with 10% human serum (male nontransfused), 1% PHA (Gibco), and recombinant IL-2 ($4 \times 10^6$ U/mg, courtesy of Biotest Pharma, Dreieich, Federal Republic of Germany). Contents of positive wells were transferred to 96-well microtiter plates containing fresh medium and $10^5$ stimulators between days 7 and 11, and to 24-well cluster plates with $5 \times 10^5$ stimulators between days 12 and 16. Cultures were given fresh medium every 3 or 4 days and fresh stimulators every 3 weeks thereafter.

### Surface-Marker Phenotyping

Cells preincubated with Intraglobin were labeled with mAb for CD2 (OKT11), CD3 (OKT3), CD4 (OKT4), and CD8 (OKT8) from the ATCC. MAb for CD45RA (IOL2), CD45RO (UCHL1), and CD16 (ION16) were purchased from Dianova GmbH, Hamburg, and CD56 (Leu19) was from Becton-Dickinson (Erembodegem, Belgium). The TCR$\delta$1 mAb reacting with nonpolymorphic determinants of the TCR$\delta$ chain, and the WT31 mAb reacting with TCR components predominantly of $\alpha/\beta$-TCR were kind gifts of Dr. M. Brenner, Boston, and Dr. W. Tax, Nijmegen, the Netherlands, respectively. MAb specific for TCR V$\beta$ families were C37 [V$\beta$5.3 (Posnett et al., 1988)], OT145 [V$\beta$6.7 (Posnett et al., 1986)], Mx9 [V$\beta$8 (Carrel et al., 1986)], and 3D6 [V$\beta$5 (Lipoldova et al., 1989)], kindly donated from the producing laboratories. The class I mAb W6/32 was used as a positive control (W6/32.HL) and its nonbinding variant as a negative control (W6/32.HK). CD5 was detected using local reagent TÜ71, and CD25 (IL-2-receptor $\alpha$ chain) using TÜ69. Labeled cells were developed with FITC-conjugated F(ab)2 rabbit anti-mouse IgG and analyzed on a FACS IV. Dead cells were excluded by electronic gating and fluorescence histograms were area-corrected to 10,000 cells.

### Northern and Southern Blotting for TCR Analysis

For Northern blot analysis, total cellular RNA was prepared by extraction with 4 M guanidinium isothiocyanate (GI) and ultracentrifugation through a CsCl cushion (Chirgwin et al., 1979). Cells were washed twice in cold PBS before lysis. Each RNA sample was quantified spectrophotometrically by absorbance at 260 and 280 nm. Approximately 12 $\mu$g of each total RNA sample was analyzed by electrophoresis on 1% (w/v) agarose, 2.2 M (w/v) formaldehyde gels. Ethidium bromide (1 $\mu$g/ml) was added to each sample to visualize the ribosomal RNAs. Following electrophoresis, RNAs were transferred to positively charged nylon membranes (Hybond $N^+$, Amersham, UK) and fixed with 0.05 M NaOH for 5 min.

Human cDNA probes were labeled with $\alpha^{32}$P-dATP (ca. 110 TBq/mmol, Amersham) by the random primer method as described by Feinberg and Vogelstein (1983) to a specific activity of ca. $10^9$ cpm/$\mu$g. pGA5, a kind gift of Dr. D. Mathieu-Mahul, Paris, is specific for the TCR$\alpha$ chain and originally derived from the ALL line HPB-MLT (Sim et al., 1984). This pUC13 subclone contains the 1.3-kb EcoRI fragment spanning the V-J-C region and part of the 3'-untranslated region. The TCR$\beta$-chain-specific cDNA probe pC$\beta$REX (Acuto et al., 1985) was from Dr. H.D. Royer (Heidelberg) and consists only of C-region sequences (800 bp EcoRI×BglII in pBR322). The cDNA probe specific for the TCR$\gamma$ chain was kindly provided by Dr. T.W. Mak, Ontario, as a 1.6-kb EcoRI insert in pUC13, originally isolated from a PBMC library, and containing the V-J-C $\gamma$1 region. 0-240, a cDNA probe specific for the TCR$\delta$ chain (kind gift of Dr. M.S. Krangel, Boston) spans the C-region and 3'-untranslated region (1.5-kb EcoRI in pUC18) (Hata et al., 1987).

Hybridization was performed overnight at 65°C in a solution containing 1% BSA, 1 mM $Na_2$-EDTA, 0.5 M $Na_2$ $HPO_4$ at pH 7.2 and 7% SDS, according to a method first described by Church and Gilbert (1984). For rehybridization, the membrane was stripped by washing in 0.1% SDS at 95°C for 3 to 5 min.

For Southern blot analysis, high-molecular-weight genomic DNA was prepared from cell lines and T-cell clones by standard techniques. Briefly, the cell lysates were digested with proteinase K and DNA was then purified by extraction with phenol and precipitation with ethanol. DNA (5–7.5 $\mu$g) of each sample was digested with EcoRI, BamHI, or HindIII (5 U/$\mu$g DNA), size-fractionated on 0.6 to 0.7% agarose gels and further treated according to Southern (Southern, 1975). The DNA was fixed on Hybond $N^+$ by alkali treatment (0.4 M NaOH, 20 min). Labeling and hybridization procedures were performed as for Northern blotting. The same TCR$\beta$ probe was also used for both. For analysis of TCR$\gamma$-chain rearrangement, a J-region-specific probe (pH60) was used (kind gift of Dr. T.H. Rabbitts, Cambridge). This subclone (700-bp HindIII× EcoRI in pUC8 [Lefranc and Rabbitts, 1985] was originally derived from the genomic phage M13H60.

### Proliferation

1×$10^4$ cloned cells were incubated with stimulating agents in round-bottom microtiter plates for different periods of time, in RPMI medium supplemented with 10% human serum. Proliferation was quantified by measuring nuclear incorporation of tritiated thymidine ($^3$H-TdR, Amersham-Buchler, Braunschweig, specific activity 185 GB/q/mmol used at 37 kBq/well) after a 14-hr pulse label. Radioactivity was measured by liquid scintillation counting and data reduction followed using in-house programs (Schaudt and Pawelec, 1991). The stimulating agents used were medium alone or the following human lymphokines: IL-2 (Lymphocult T, Biotest, Frankfurt; activity standardized against the WHO-BRMP-NIH control), IL-4 (kind gift of Dr. M. Schreier, Sandoz, Basel; specific activity 1×$10^8$ U/mg protein), IL-3 and GM-CSF (courtesy of Drs. F. Seiler and B. Krumwieh, Behring-Werke, Marburg; both at ca. $10^7$ U/mg protein), and IL-7 (purchased from Biosource International, Westlake Village, CA; specific activity 1× $10^7$ U/mg protein), or PHA (Gibco), B-lymphoblastoid cell lines (B-LCL) expressing various HLA class II antigens including HLA-Dw 1-10, 12, 14, and "HAG." Mouse L cells transfected with human HLA-DR genes were also used as stimulators in some experiments. These transfectants were obtained through the 11th International Histocompatibility Workshop.

In some experiments, attempts were made to block autocrine proliferation with neutralizing antibodies against IL-1$\alpha$, IL-1$\beta$, or IL-2 (all affinity-purified goat anti-human IgG from British Biotechnology), or with a CD25 mAb (local reagent TÜ69).

### Cytotoxicity

Standard $^{51}$Cr-release assays were employed to measure cell-mediated cytotoxicity. 2–4×$10^6$ target cells were incubated with 3.7×$10^6$ kBq of sodium $^{51}$chromate (Amersham) for 90 min at 37°C, followed by repeated washing and use at 2 ×$10^3$ cells/well. Experiments were carried out in U-bottom microtiter plates with at least four different ratios of effector to target cells. After 4 hr, cell-free supernatant was collected and radioactivity quantified in a $\gamma$ counter. Spontaneous $^{51}$Cr release and maximum release were determined in each experiment. The data were collected and reduced to a single-value expression of cytolytic activity using the lytic units calculation (Schaudt and Pawelec, submitted for publication), where one LU was defined as the number of effectors per $10^7$ cells resulting in 25% specific isotope release from 2000 target cells.

### Cytokine Secretion

Cloned cells and 80 Gy-irradiated B-LCL were cocultured at $5\times10^5$/ml in 16-mm-diameter culture plate wells for 24 or 48 hr with or without 1% PHA. Cell-free supernatants were collected for cytokine bioassay and immunoassay. Granulocyte/macrophage colony-stimulating factor (GM-CSF) was measured using an in-house ELISA with reagents from Genzyme: microtiter plates were coated with anti-GM-CSF catching antibody overnight at 4°C and the remaining sites blocked with 1% bovine serum albumin for 1 hr. Test samples and standard GM-CSF controls were incubated at room temperature for 90 min, followed by addition of anti-GM-CSF serum and visualization using the peroxidase-conjugated goat anti-rabbit $F(Ab)_2IgG$ and phenylenediamine/$H_2O_2$ solution. The sensitivity of the assay was approximately 0.1 U/ml (where one unit is the amount required to generate one colony [50 cells] from $7.5\times10^4$ normal human BM cells in soft agar over 14 days). There was no cross-reactivity with IL-3 or G-CSF. Tumor necrosis factor (TNF-$\alpha$) was quantified using a commercial IRMA from Medgenix (Düsseldorf, FRG), with a sensitivity of 10 pg/ml and no cross-reactivity on TNF-$\beta$, IL-1, IL-2, or interferons. T-cell growth factors were measured in bioassays by titrating test supernatants onto factor-dependent T-cell lines, and assaying proliferation compared to titrated amounts of standard IL-2. Although not proven, it is assumed that the majority of the proliferation observed is caused by IL-2.

### Detection of Cytokines at the mRNA Level

RNA was isolated from cloned cells by a modification of the method of Chomczynski and Sacchi (1987) after stimulation with 1% PHA and 80 Gy-irradiated LCL for 5 hr. Briefly, cells were lysed in guanidinium isothiocyanate buffer (4 M GI, 20 mM sodium acetate at pH 5.2, 0.1 mM dithiothreitol, and 0.5% N-laurylsarcosine). Then 2 M sodium acetate (pH 4.0), water-saturated phenol, and chloroform:isoamyl alcohol (49:1) were sequentially added, vortexed, and chilled on ice for 15 min. Phases were separated by centrifugation at 10,000 g for 20 min at 4°C. RNA was precipitated from the aqueous supernatant with isopropyl alcohol, washed with 70% ethanol, dried, and dissolved in water. 0.5 $\mu$g of total RNA was reverse transcribed by addition of 0.1 by volume of 10×PCR reaction buffer (100 mM Tris-HCl at pH 8.3, 500 mM KCl, 15 mM $MgCl_2$, and 0.01% gelatine), 0.5 $\mu$g oligo-dT-Primer (Gibco-BRL), 0.2 mM of each deoxynucleotide (Perkin-Elmer Cetus, Norwalk, CT) 20 U rRNasin RNase inhibitor (Promega), and 200 U of Moloney-murine leukemia virus reverse transcriptase (Gibco-BRL). The reaction proceeded at 42°C for 1 hr and was terminated by heating to 95°C for 5 min.

Eighty microliters of 1×PCR-buffer containing 20 pmol each of upstream and downstream primer and 1 U of Taq DNA polymerase (Perkin-Elmer Cetus) were added to the 20 $\mu$l of reverse transcriptase reaction mixture and overlaid with mineral oil. The reaction was carried out in a Bio-Med Thermocycler 60 (Bachofer, FRG) with the following cycling parameters: 1 min at 95°C (denaturation), 1 min at 60°C (annealing), and 2 min at 72°C (extension) for 30 cycles, followed by an additional 5 min at 72°C. To analyze the specific sizes of the amplification products, 20-$\mu$l aliquots were electrophoresed on 2% agarose gels, stained with ethidium bromide, and compared with molecular-weight standards.

The sequences of the primers used to detect the mRNA transcripts coding for IL-3, IL-4, IFN-$\gamma$, GM-CSF, and $\beta$-actin were taken from Ehlers and Smith (1991), and for IL-2 from Wieder et al. (1990). The primer pair for IL-9 was deduced from the published human IL-9 sequence (Yang et al., 1989) for which the sense primer corresponded to nucleotide 9-29 (5'-CTGTCAAG-ATGCTTCTGGCCA-3') and the antisense primer to 363-384 (5'-GTCAGCGCGTTGCCTGCCGTGG-3'). The specificity of the amplification process was determined by Southern blot analysis of the derived fragment using a 489-bp EcoRI×HindIII cDNA probe, a kind gift of Dr. J. van Snick, Brussels (Renauld et al., 1990). All primers were synthesized on a Pharmacia Gene Assembler Plus and purified by NAP™10 columns (Pharmacia LKB, Sweden).

### Suppression

Cloned cells were irradiated at 20 Gy and titrated into MLC between autologous or allogeneic PBMC as responding cells and a 30 Gy-irradiated pool (>20 donors) as stimulators. MLC consisted of $5\times10^4$ responders and an equal number of stimulators in round-bottom microtiter plates with medium containing 10% human serum. After 5 days, 37-kBq $^{3}$H-TdR/well were added and cultures harvested for $\beta$-scintillation counting 14 hr later. Controls consisted of responder PBMC alone, and MLC in the absence of added cloned cells.

### ACKNOWLEDGMENTS

This work was supported in part by grants from the Deutsche Forschungsgemeinschaft (SFB 120/A1 and Pa 361/1-1) and from the Deutsche Krebshilfe (W2/91/Pa1). The assistance of David Vöhringer is gratefully acknowledged. We thank Drs. D. Mathieu-Mahoul, Paris, for pGA5; H.D. Royer, Heidelberg, for pC$\beta$-REX; T. Mak, Ontario, and T.H. Rabbitts, Cambridge, for the TCR$\gamma$ chain cDNAs; M. Krangel, Boston, for 0-240; and J. van Snick, Brussels, for the IL-9 cDNA probe. We thank Drs. U. Schwuléra, Frankfurt, for IL-2; F. Seiler and D. Krumwieh, Marburg, for IL-3 and GM-CSF; and M. Schreier, Basel, for IL-4. We are grateful to Drs. A. Boylston, Leeds, D. Posnett, New York, M. Brenner, Boston, W. Tax, Nijmegen, and S. Carrel, Lausanne, for mAb. We also thank Dr. Ø. Bruserud and Prof. C.A. Müller for critically reading the manuscript.

*(Received August 5, 1992)*

*(Accepted December 10, 1992)*

### REFERENCES

Abromson-Leeman S.R., Jayaraman S., and Dorf M.E. (1990). Characterization of T cell clones from an athymic mouse. J. Immunol. **144:** 2451–2458.

Acuto O., Campen T.J., Royer H.-D., Hussey R.E., Poole C.B., and Reinherz E.L. (1985). Molecular analysis of T cell receptor (Ti) variable region (V) gene expression: Evidence that a single Ti beta V gene family can be used in formation of V domains on phenotypically and functionally diverse T cell populations. J. Exp. Med. **161:** 1326–1343.

Barcena A., Sanchez M.J., Delapompa J.L., Toribio M.L., Kroemer G., and Martinez C. (1991). Involvement of the interleukin-4 pathway in the generation of functional gammadelta-T-cells from human pro-T cells. Proc. Natl. Acad. Sci. USA **88:** 7689–7693.

Benveniste P., Chadwick B.S., Miller R.G., and Reimann J. (1990). Characterization of cells with T cell markers in athymic nude bone marrow and of their *in vitro*-derived clonal progeny: Comparison with euthymic bone marrow. J. Immunol. **144:** 411–419.

Bertho J.M., Mossalayi M.D., Dalloul A.H., Mouterde G., and Debre P. (1990). Isolation of an early T-cell precursor (CFU-TL) from human bone marrow. Blood **75:** 1064–1068.

Carrel S., Isler P., Schreyer M., Vacca A., Salvi S., Giuffre L., and Mach J.-P. (1986). Expression on human thymocytes of the idiotypic structures (Ti) from two leukemic T cell lines Jurkat and HPB-ALL. Eur. J. Immunol. **16:** 649–654.

Carrera A.C., Rincón M., Sánchez-Madrid F., López-Botet M., and de Landazuri M.O. (1988). Triggering of co-mitogenic signals in T cell proliferation by anti-LFA-1 (CD18, CD11a), LFA-3, and CD7 monoclonal antibodies. J. Immunol. **141:** 1919–1924.

Chirgwin J.M., Przybyla A.E., MacDonald R.J., and Rutter W.J. (1979). Isolation of biologically active ribonucleic acid from sources enriched in ribonuclease. Biochemistry **1894:** 5294–5299.

Chomczynski P., and Sacchi N. (1987). Single-step method of RNA isolation by acid guanidinium thiocyanate-phenol-chloroform extraction. Anal. Biochem. **162:** 156–159.

Church G.M. and Gilbert W. (1984). Genomic sequencing. Proc. Natl. Acad. Sci. USA **81:** 1991–1995.

Costantinides Y., Kingsley G., Pitzalis C., and Panayi G.S. (1991). Inhibition of lymphocyte proliferation by a monoclonal antibody (RFT2) against CD7. Clin. Exp. Immunol. **85:** 164–167.

Ehlers S., and Smith K.A. (1991). Differentiation of T cell lymphokine gene expression: The *in vitro* acquisition of T cell memory. J. Exp. Med. **173:** 25–36.

Emara M., Baldwin W.M. III, Finn O.J., and Sanfilippo F. (1989). A human suppressor T-cell factor that inhibits T-cell replication by interaction with the IgM-Fc receptor (CD7). Hum. Immunol. **25:** 87–102.

Feinberg A.P., and Vogelstein B. (1983). A technique for radiolabeling DNA restriction endonuclease fragments to high specific activity. Anal. Biochem. **132:** 6–13.

Hallet M.-M., Peyrat M.-A., Moisan J.-P., Soulillou J.-P., and Moreau J.-F. (1989). Proliferation dependence and production of IL-2 in human alloreactive T-cell clones. Cell. Immunol. **124:** 95–106.

Hata S., Brenner M.B., and Krangel M.S. (1987). Identification of putative human T cell receptor delta complementary DNA clones. Science **238:** 678–682.

Haynes B.F., Martin M.E., Kay H.H., and Kurtzberg J. (1988). Early events in human T cell ontogeny. Phenotypic characterisation and immunohistologic localisation of T cell precursors in early human fetal tissues. J. Exp. Med. **168:** 1061–1071.

Hori T., Malefyt R.D., Duncan B.W., Harrison M.R., Roncarolo M.G., and Spits H. (1991). Cloning of a novel cell type from human fetal liver expressing cytoplasmic CD3-delta and CD3-epsilon but not membrane CD3. Int. Immunol. **3:** 353–357.

Jung K.L.J., Roy A.M., and Chakkalath H.R. (1992). CD7 augments T cell proliferation via the interleukin 2 autocrine pathway. Cell. Immunol. **141:** 189–199.

Lecron J.C., Mossalayi M.D., Tanzer J., Gombert J., and Goube de Laforest P. (1989). Signal requirements for the generation of T-lymphocytes from T-depleted bone marrow cells: A sequential study. Exp. Hematol. **17:** 785–790.

Lefranc M.P., and Rabbitts T.H. (1985). Two tandomly organised human genes encoding the T cell gamma constant region sequences show multiple rearrangement in different T cell types. Nature **316:** 464–466.

Lipoldova M., Boylston A.W., Yssel H., and Owen M.J. (1989). T-cell receptor V$\beta$5 usage defines reactivity to a human T-cell receptor monoclonal antibody. Immunogenetics **30:** 162–168.

Lobach D.F., Hensley L.L., Ho W., and Haynes B.F. (1985). Human T cell antigen expression during the early stages of fetal thymic maturation. J. Immunol. **135:** 1752–1759.

Moreau J.-F., and Miller R.G. (1983). Growth at limiting dilution of human T cell colonies from T cell-depleted peripheral blood leukocytes. J. Immunol. **130:** 1139–1145.

Mossalayi M.D., Dalloul A.H., Bertho J.-M., Lecron J.-C., De

Laforest P.G., and Debré P. (1990). *In vitro* differentiation and proliferation of purified human thymic and bone marrow $CD7^{+}CD2^{-}$ T-cell precursors. Exp. Hematol. **18:** 326–331.

Mossalayi M.D., Goube de Laforest P., Guilhot F., Kallil G., Ntyame E., Larroque V., Fellous M., and Tanzer J. (1985). Agar human T cell colony growth promoted by a B+ null cell-derived lymphokine distinct from IL-2. J. Immunol. **134:** 2400–2404.

O'Flynn K., Krensky A.M., Beverley P.C.L., Burakoff S.J., and Linch D.C. (1985). Phytohaemagglutinin activation of T cells through the sheep red blood cell receptor. Nature **313:** 686–687.

Palathumpat V., Dejbakhshjones S., Holm B., Wang H., Liang O., and Strober S. (1992). Studies of CD4– CD8– alphabeta-bone marrow T-cells with suppressor activity. J. Immunol. **148:** 373–380.

Pantaleo G., Zocchi M.R., Ferrini S., Poggi A., Tambussi G., Bottino C., Moretta L., and Moretta A. (1988). Human cytolytic cell clones lacking surface expression of T cell receptor $\alpha/\beta$ or gamma/$\delta$. Evidence that surface structures other than CD3 or CD2 molecules are required for signal transduction. J. Exp. Med. **168:** 13–24.

Posnett D.N., Gottlieb A., Bussel J.B., Friedman S.M., Chiorazzi N., Li Y., Szabo P., Farid N.R., and Robinson M.A. (1988). T cell antigen receptors in autoimmunity. J. Immunol. **141:** 1963–1969.

Posnett D.N., Wang C.I., and Friedman S.M. (1986). Inherited polymorphism of the human T-cell antigen receptor detected by a monoclonal antibody. Proc. Natl. Acad. Sci. USA **83:** 7888–7892.

Preffer F.I., Kim C.W., Fischer K.H., Sabga E.M., Kradin R.L., and Colvin R.B. (1989). Identification of pre-T cells in human peripheral blood. Extrathymic differentiation of $CD7^{+}CD3^{-}$ cells into $CD3^{+}$ gamma/$\delta^{+}$ or $\alpha/\beta^{+}$ T cells. J. Exp. Med. **170:** 177–190.

Renauld J.C., Goethals A., Houssiau F., Van Roost E., and Van Snick J. (1990). Cloning and expression of a cDNA for the human homolog of mouse T cell and mast cell growth factor P40. Cytokine **2:** 9–12.

Sarawar S.R., Yang C.P., and Bell E.B. (1991). T-cell receptor-bearing cells from athymic nude rats respond to alloantigen *in vitro* but are defective *in vivo*. Immunology **73:** 334–341.

Schaudt K., and Pawelec G. (1991). Computerised transfer and processing of data from experiments measuring cellular proliferation by incorporation of tritiated thymidine. J. Immunol. Methods **138:** 155–164.

Shimizu Y., Van Seventer G.A., Ennis E., Newman W., Horgan K.J., and Shaw S. (1992). Cross linking of the T-cell-specific accessory molecules CD7 and CD28 modulates T-cell adhesion. J. Exp. Med. **175:** 577–582.

Sim G.K., Yagüe J., Nelson J., Marrack P., Palmer E., Augustin A., and Kappler J. (1984). Primary structure of human T cell receptor alpha chain. Nature **312:** 771–775.

Southern E.M. (1975). Detection of specific sequences among DNA fragments separated by gel electrophoresis. J. Mol. Biol. **98:** 503–517.

Triebel F., Robinson W.A., Hayward A.R., and Goube de Laforest P. (1981). Characterisation of the T lymphocyte colony forming cells and evidence for the acquisition of T cell markers in the absence of the thymic microenvironment in man. J. Immunol. **126:** 2020–2023.

Watanabe Y., Sudo T., Minato N., Ohnishi A., and Katsura Y. (1991). Interleukin-7 preferentially supports the growth of gammadelta T-cell receptor-bearing T-cells from fetal thymocytes *in vitro*. Int. Immunol. **3:** 1067–1075.

Wieder K.J., Walz G., Zanker B., Sehajpal P., Sharma V.K., Skolnik E., Strom T.B., and Suthanthiran M. (1990). Physiologic signaling in normal human T-cells: mRNA phenotyping by northern blot analysis and reverse transcription-polymerase chain reaction. Cell. Immunol. **128:** 41–51.

Yang Y.-C., Ricciardi S., Ciarletta A., Calvetti J., Kelleher K., and Clark S.C. (1989). Expression cloning of a cDNA encoding a novel human hematopoietic growth factor: Human homologue of murine T-cell growth factor P40. Blood **74:** 1880–1884.

